# Edible Mushrooms as Novel Myco-Therapeutics: Effects on Lipid Level, Obesity, and BMI

**DOI:** 10.3390/jof8020211

**Published:** 2022-02-21

**Authors:** Faheem Mustafa, Hitesh Chopra, Atif Amin Baig, Satya Kumar Avula, Sony Kumari, Tapan Kumar Mohanta, Muthupandian Saravanan, Awdhesh Kumar Mishra, Nanaocha Sharma, Yugal Kishore Mohanta

**Affiliations:** 1 School of Health Sciences, University of Management and Technology, Lahore 54782, Pakistan; faheem.mustafa@umt.edu.pk; 2 Unit of Biochemistry, Faculty of Medicine, Universiti Sultan Zainal Abidin, Kuala Terengganu 20400, Malaysia; atifamin@unisza.edu.my; 3 Chitkara College of Pharmacy, Chitkara University, Punjab 140401, India; chopraontheride@gmail.com; 4 Natural and Medical Sciences Research Centre, University of Nizwa, Nizwa 616, Oman; chemisatya@unizwa.edu.om (S.K.A.); tapan.mohanta@unizwa.edu.om (T.K.M.); 5 Department of Applied Biology, School of Biological Sciences, University of Science and Technology Meghalaya, Ri-Bhoi 793101, India; sonykumari_15@yahoo.com; 6 AMR and Nanotherapeutics Laboratory, Department of Pharmacology, Saveetha Dental College, Saveetha Institute of Medical and Technical Sciences, Chennai 600077, India; bioinfosaran@gmail.com; 7 Department of Biotechnology, Yeungnam University, Gyeongsan 8541, Gyeongsangbuk-do, Korea; 8 Institute of Bioresources and Sustainable Development, Department of Biotechnology, Government of India, Imphal 795001, India

**Keywords:** edible mushroom, obesity, lipid profile, body mass index, gut microbiota, anti-obesity agent, dietary habit

## Abstract

Obesity, usually indicated by a body mass index of more than 30 kg/m^2^, is a worsening global health issue. It leads to chronic diseases, including type II diabetes, hypertension, and cardiovascular diseases. Conventional treatments for obesity include physical activity and maintaining a negative energy balance. However, physical activity alone cannot determine body weight as several other factors play a role in the overall energy balance. Alternatively, weight loss may be achieved by medication and surgery. However, these options can be expensive or have side effects. Therefore, dietary factors, including dietary modifications, nutraceutical preparations, and functional foods have been investigated recently. For example, edible mushrooms have beneficial effects on human health. Polysaccharides (essentially β-D-glucans), chitinous substances, heteroglycans, proteoglycans, peptidoglycans, alkaloids, lactones, lectins, alkaloids, flavonoids, steroids, terpenoids, terpenes, phenols, nucleotides, glycoproteins, proteins, amino acids, antimicrobials, and minerals are the major bioactive compounds in these mushrooms. These bioactive compounds have chemo-preventive, anti-obesity, anti-diabetic, cardioprotective, and neuroprotective properties. Consumption of edible mushrooms reduces plasma triglyceride, total cholesterol, low-density lipoprotein, and plasma glucose levels. Polysaccharides from edible mushrooms suppress mRNA expression in 3T3-L1 adipocytes, contributing to their anti-obesity properties. Therefore, edible mushrooms or their active ingredients may help prevent obesity and other chronic ailments.

## 1. Introduction

Obesity is an illness in which the amount of body fat is elevated. Hence, it increases the risk of other illnesses such as diabetes, hypertension, and CVD. Multiple factors, notably heredity, nutrition, environment, lifestyle, and infectious agents are the main causes of obesity [1]. Obesity is a complex disease characterised by excessive fat accumulation. Heftiness is beyond the actual issue. It is an ailment that builds the danger of different illnesses, such as coronary illness, diabetes, hypertension, and a few types of cancer. Obesity is a multifactorial metabolic ailment characterised by secondary complications, gut epithelial hyperpermeability, and dysregulation of digestive microbiota. It has become a global issue due to the consumption of high-fat food and lack of sufficient physical activity worldwide. It causes increased incidences of lifestyle disorders such as type 2 diabetes, cardiovascular illnesses, and cancer, usually resulting in reduced lifespan [2]. Safe, affordable, and extensively accessible anti-obesogenic methods are needed to address obesity and its consequences. Weight is fundamentally a disparity between energy intake and its expenditure. A weight loss of 5% further leads to medical issues and decreases the likelihood of cardiovascular sickness and type 2 diabetes mellitus (T2DM) [3]. Although a low-calorie diet combined with continuous standard exercise prompts weight loss and has been the most effective strategy for forestalling and treating obesity, it is challenging to execute. It has inconsistent success owing to the adaptive processes that conserve energy stored in the body. Several anti-obesogenic pharmaceuticals have also been licenced for use. When taken for a year, orlistat, the most commonly used long-term anti-obesogenic medication, reduces body weight by 3% on average. However, it may cause gastrointestinal side effects, subacute liver failure, and acute renal damage. Surgery for weight loss via gastric bypass or gastric banding is more successful than anti-obesogenic medications. However, the procedure is expensive, physically intrusive, and not suitable for most people who are overweight.

Mushrooms are spore-bearing fruiting bodies of fungi that grow above the ground. They are rich in starches and proteins but are a poor source of fat [4]. Many researchers have reported the nutritional value of various mushrooms. Reis et al. reported the composition of *Agaricus bisporus* as 14.1% protein, 2.2% fat, and 74.0% carbohydrates, while another mushroom *Pleurotus ostreatus* contains 7.0% protein, 1.4% fat, and 85.9% carbohydrates [5]. Mushrooms also contain micronutrients, mainly various types of vitamin B such as riboflavin, niacin, and pantothenic acid [6]. The consumption of 100 g of mushrooms provide 22 calories. Oyster mushrooms are common in South Asian countries. They are used to make oyster sauce in Chinese cuisine. The cremini mushroom is also known as the baby Bella mushroom. The portobello mushroom is mainly used for highly woody flavours and has immunomodulatory properties [7]. Aromatic shiitake mushrooms in Italian foods have antiviral properties [8]. Maitake mushrooms have immune-protective and anti-tumour properties [9,10]. The pioppino mushroom (*Cyclocybe aegerita*) is a good source of nutrients (amino acids, malic acid, and sugars) and has anticancer, antifungal, and antiviral properties [11,12].

Mushrooms are used as food and nutraceuticals. They are essential nutrient supplements that play a vital role in health and illnesses. They have low polyunsaturated fat. Therefore, eating mushrooms helps to reduce weight, as a low fat, low glucose, and high mannitol diet can prevent diabetes [13]. Mushrooms also have low sodium and no cholesterol, which prevents hypertension [14]. Mushrooms have high levels of antioxidants. Few researchers have reported their preventive effect against cancer [15,16]. Mushrooms possess antioxidant properties, which aids in the antioxidant defence mechanisms of cells [17]. They have anti-inflammatory properties and reduce the risk of obesity-related dyslipidaemia and hypertension [7,8,18,19,20,21,22,23,24,25,26,27,28]. Mushroom consumption on a regular basis is useful in curing metabolic disorders that include obesity. Therefore, they could be nutraceuticals of choice in the future for anti-obesity treatment. *P. ostreatus*, frequently called the oyster mushroom, is one of the world’s most widely consumed mushrooms after white button mushrooms (*A. bisporus*). *P. ostreatus* is especially significant since it can colonise and make use of a broad range of lignocellulosic substrates from natural deposits. It grows more rapidly than other edible mushrooms. In addition, *P. ostreatus* contains bioactive substances, including β-glucans, which aid in cardiometabolic health [29,30]. *P. ostreatus* has two-fold more β-glucan content compared to *A. bisporus*. They are nutritional fibres that have gained popularity due to their ability to reduce insulin obstruction, hypertension, dyslipidaemia, and obesity. β-glucans are exceptionally good supplements for human gastrointestinal health, and their fermentation is believed to contribute to the wellbeing of the intestine. These effects have been widely reported in studies with oat and grain β-glucans. Mevinolin, also known as lovastatin, has an inhibitory effect on 3-hydroxy-3-methylglutaryl coenzyme A (HMG-CoA) reductase and is also involved in decreasing cholesterol synthesis. In addition, in vitro digestion of *P. ostreatus* produces bioactive peptides that inhibit angiotensin-converting enzymes [31]. *P. ostreatus* contains abundant phenolic compounds which may be involved in lowering the blood pressure [32,33]. *P. ostreatus* has been used in animal studies and showed hypoglycaemic, hypolipidemic, and antioxidant effects. Animals consuming *P. ostreatus* exhibited reduced food intake and weight gain, suggesting the anti-obesogenic potential of this edible mushroom [32,33,34,35,36,37].

**Figure 1:**
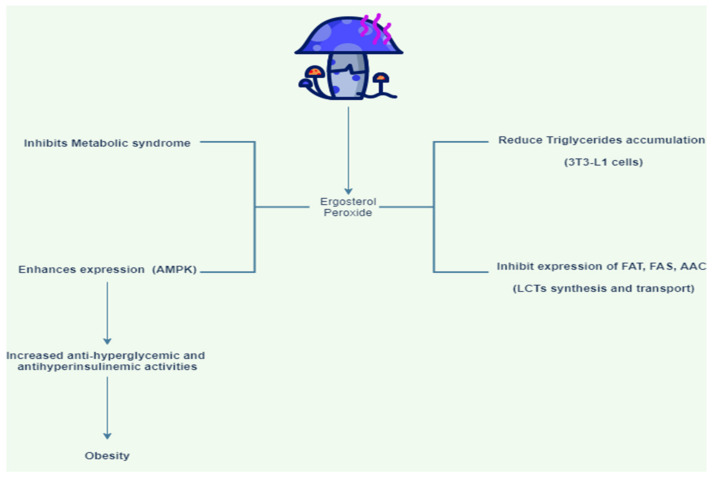
Pharmacological effects of ergosterol peroxide derived from mushrooms on obesity [38]. Ergosterol shows anti-obesity effect by reducing triglycerides accumulation, inhibiting expression of FAT, FAS, AAC, inhibiting metabolic syndrome, enhancing AMPK expression, increasing antihyperglycemic, and anti-hyperinsulinemic activities.

Ergosterol peroxide is a compound found in mushrooms that decreases the accumulation of fatty acids in 3T3-L1 cells (Figure 1) [38]. This compound inhibits the mRNA upregulation of sterol regulatory element binding protein-1c (SREBP-1c). SREBP-1c is a sterol response limiting protein that regulates the response of sterol in the body. In addition, ergosterol peroxide treatment inhibits the expression of unsaturated fat synthase, unsaturated fat translocase, and acetyl-coenzyme A carboxylase involved in the synthesis and transportation of long-chain unsaturated fatty acids. Since it aids in the prevention of obesity and related metabolic conditions, these reports suggest that ergosterol peroxide obtained from *G. lucidum* might be a potential drug for anti-obesity treatment [38]. AMP-activated protein kinase (AMPK) is a key regulator of homeostasis. Increased AMPK activity showed antihyperglycemic and anti-hyperinsulinemic effects which resulted in reduced obesity in mice. Consumption of *H. erinaceus* (a mushroom) powder reduced total plasma cholesterol and leptin levels in mice that were fed a diet containing the amount of fat tissue [39].

*G. lucidum* has anti-diabetic properties and has been used in conventional Chinese medicine. In mice following a high-fat diet (HFD), administration of water concentrate of *G. lucidum* mycelium (WEGL) reduced bodyweight, irritation, and insulin obstruction [40]. Along with reducing HFD-induced gut dysbiosis (as seen by lower Firmicutes-to-Bacteroidetes ratio and increased abundance of endotoxin producing proteobacteria), WEGL administration alleviates metabolic endotoxemia [41]. The weight-reducing and microbiota-regulatory effects can be passed on from WEGL-treated mice to HFD-administered ones by faecal exchange. In addition, high molecular weight polysaccharides (>300 kDa) present in the WEGL have shown anti-obesity and microbiota-regulating properties. *G. lucidum* and its high atomic weight polysaccharides can be used as prebiotics in overweight individuals to treat gut dysbiosis and metabolic disorders [40].

*Pleurotus citrinopileatus* is another potential source of bioactive mixtures and therefore, can be used in anti-obesity treatment [42,43,44]. One study assessed the anti-obesity and hypolipidemic effects of *P. citrinopileatus* water extract (PWE) in high-fat diet-induced obese (DIO) C57BL/6J mice. They were administered with PWE in gradually increasing concentrations (400 to 800 mg/kg of body weight, independently) along with a high-fat diet for 12 weeks [45]. Within 12 weeks, the weight gain, fat build-up, and food utilisation of DIO mice were drastically reduced in mice administered with PWE. PWE also decreased fatty acid, cholesterol, and low-density lipoprotein levels in the blood, simultaneously increasing the activity of aspartate transaminase, non-esterified unsaturated fats, creatinine levels, and high-density lipoprotein levels. Moreover, PWE also enhanced glucose tolerance in HFD mice and showed a high potential for managing obesity and other metabolic diseases [45].

The focus of this review is the aetiology and pathophysiology of overweight and the anti-obesity effects of edible mushrooms. We will discuss the effect of consumption of mushrooms on food processing, food intake, reducing food craving, energy consumption, lipid accumulation, and gut microbiota.

Relevant studies pertaining to the application of mushrooms for anti-obesity effects were selected from several scientific databases such as Google Scholar (http://www.scholar.google.co.in) (accessed on 10 December 2021), PubMed (http://www.ncbi.nlm.nih.gov/pubmed) (10 December 2021), Elsevier (https://www.elsevier.com/en-in), ScienceDirect (http://www.sciencedirect.com) (accessed on 10 December 2021), Springer (http://www.springer.co.in) (accessed on 10 December 2021), and Scopus (http://www.scopus.com) (accessed on 10 December 2021). Publications that had the full text available and book chapters in English were only reviewed.

## 2. Aetiology of Obesity

Obesity is not an acute condition that develops instantly. It takes time to develop. It is a chronic condition and its development results from a complex interaction between an individual’s genetics and environment. Leptin and ghrelin hormones regulate energy homeostasis and obesity develops due to a long-term imbalance in the energy homeostasis. A sedentary lifestyle and low metabolic rate trigger the onset of obesity [46,47]. Several sociocultural factors accelerate the development of obesity. They include ready access to abundant foods, low physical activity, and mechanisation [48,49].

Genetics significantly influence the development of obesity. However, the exact mechanism responsible for this development is not yet known. Genetic causes could not be easily identified through pedigree analyses. The effect of genotype is generally reduced by non-genetic factors. The tendency to gain weight is familial. Additionally, the tendency to gain weight also depends on dietary habits and lifestyle [50,51,52].

Some sociodemographic factors including age and sex also influence the development of obesity. A study conducted in Spain showed that obesity is much higher in men than in women [53]. The highest rate of obesity is observed in the 60 years age group. Level of education is also associated with obesity. Most of the epidemiological studies on obesity revealed an inverse relationship between the prevalence of obesity and level of education. Socioeconomic factors also affect obesity development [54,55,56]. Obesity is more common in advanced and well-developed countries. As adoption of Western culture is increasing in under developed countries, and the incidence of obesity is also increasing [57,58].

It is commonly known that obesity is caused due to societal changes such as reduced physical activity and dietary habits. Obesity is a multifactorial disease. Its onset occurs during childhood and adolescence. Genetics alone are not the causative agents of obesity; the environment could also result in obesity. The primary cause of obesity in majority of people is the imbalance in the consumption of energy and its use. Presently, various investigations have reported that nutrigenomics and gut microbiota are the major determinants of obesity [59,60,61,62,63].

Dietary habits are one of the primary determinants of health and diseases. It is difficult to quantify the exact portion of food to determine its health-promoting effects. In the past few decades, increasing scientific evidence has shown an association between dietary factors and chronic diseases [64,65]. These chronic diseases include cardiovascular diseases, osteoporosis, cancer, diabetes, and obesity.

## 3. Pathophysiology of Obesity

Obesity leads to several health-related problems and affects most of the body’s vital organs, resulting in serious complications. Increased food intake and lack of physical activity adversely affect the body in several ways. The initial manifestation of increased food intake is the increased triacylglycerol storage in the body’s adipose tissues. The size of fat cells increases as the body weight increases. When their maximum capacity is reached, additional adipocytes are synthesised to accommodate more triacylglycerol. When the BMI crosses 35 kg/m^2^ (or 75% higher than expected weight), it results in hypercellular obesity. One consequence of hypercellular obesity is the increased expression in lipoprotein lipase, which increases linearly with the increase in BMI up to 50 kg/m^2^. Few factors influence the storage of triacylglycerols in subcutaneous and visceral depots. Corticosteroids affect the redistribution of fat in stomach or fat-storing tissues. Fat storage in the lower body or gluteal muscles is increased at lower cortisol levels and higher oestradiol levels than testosterone. The increase in fatty acid storage is directly related to the synthesis of cholesterol. Increased level of cholesterol synthesis is related to the increased cholesterol release into the bile, which eventually results in the formation of gallstones and a nervous bladder [66,67,68,69].

## 4. Appetite Suppressing Effect of Mushrooms

**Figure 2:**
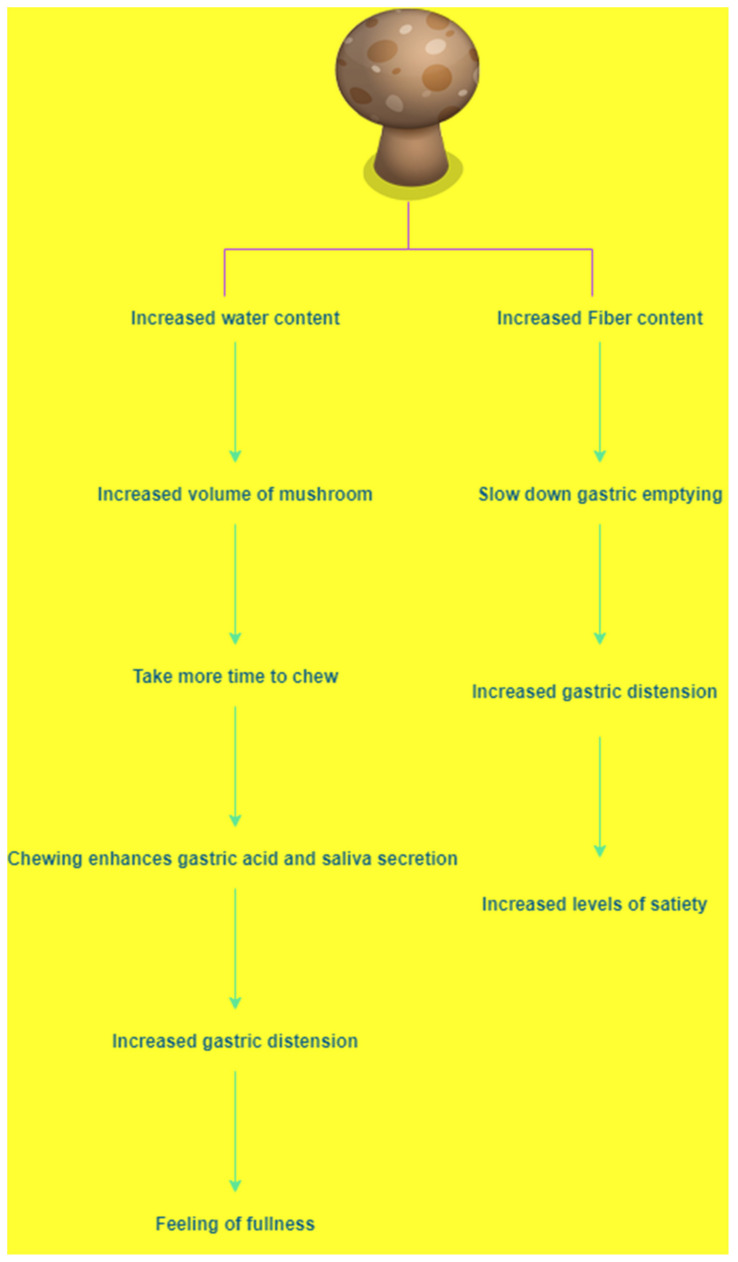
Effect of mushrooms on gastric emptying and salivary secretion.

Mushrooms are widely used in foods, medicines, and nutraceuticals. Studies have shown the effect of *A. bisporus* (white button mushroom) consumption on food intake and satiety. Mushroom-and meat-based meals have equivalent protein content. However, they differ in portion size, fibre and carbohydrate contents, and calorie intake [47]. Despite having differences in the fibre content and portion size of sandwiches made of mushrooms and meat, consumption of a mushroom sandwich was more satisfying. However, there was no effect on energy intake. In a randomised controlled investigation of 32 members who consumed two servings of meat and mushrooms for 10 days, it was shown that replacing meat with mushrooms reduced the energy intake, resulting in weight reduction [70]. Studies have shown that mushrooms have higher water content than meat. Additionally, mushrooms require a longer time to chew. Chewing enhances gastric acid and saliva secretion, which leads to increased gastric distention and gives a feeling of fullness [70]. Mushrooms also have more fibre content, which upon consumption increases the food volume in the stomach, leading to an increased satiety. Mushrooms have both fermentable and non-fermentable fibres. Depending on the type of fibres, consuming mushrooms slows down gastric emptying, enhances gastric distention, and ultimately promotes satiation (Figure 2) [70]. Additionally, it has been reported that consumption of cholesterol obtained from the root of another mushroom called thunder, reduces appetite and results in weight loss. It decreases stress of the endoplasmic reticulum and reduces leptin resistance [71].

Natural products have been used for many years to combat obesity. In this context, the appetite-suppressing effects of UP601 are well known. This standardised botanical blend of *Magnolia officinalis*, *Morus alba*, and *Yerba mate* was shown to suppress appetite in lab rats. The rats also lost weight, indicating its beneficial effects on the management of obesity and diabetes [72].

The appetite-suppressing effects of *M. alba* can be attributed to two chemical compounds isolated from its root bark: kuwanon G and albanin G. Lab rats were fed 250 and 500 mg/kg of the extract for seven days. Remarkable reduction of 58.6% and 44.8% weight loss was observed at 250 mg concentration. When the dose was increased to 500 mg, 50.1 and 44.3% weight loss was observed. Overall, total calorie intake was reduced by 20% [73]. Table 1 summarises the weight loss, anti-obesity, and hypolipidemic properties of different types of mushrooms.

## 5. Alteration of Adipocyte Function

Adipocytes are mesenchymal cells. Their essential function is to store energy in the form of lipids and protect the body from external environment. Pre-adipocytes are undifferentiated fibroblasts that can be induced to differentiate into adipocytes. A variety of hormones are produced by adipose tissue, including leptin, oestrogen, resistin, TNF and ALP, type 1 collagen, OPN, Runx2, and Ocn. The primary energy storage sites in the body are adipocytes.

Adipocytes are divided into two groups: white adipocytes, which store energy in a large and single lipid molecule and perform key endocrine functions, and brown adipocytes, which store energy in a few tiny lipid beads. However, brown adipocytes are used to generate the body heat (i.e., thermogenesis). In brown adipocytes, the activation of mitochondrial uncoupling protein 1 generates heat [108]. However, this differentiation often depends upon temperature and diet; few white adipocytes have the attributes of brown adipocytes (called brite or beige adipocytes) and *vice versa*. Adipocytes have more lipid-containing vacuoles that store fatty acids and cholesterol esters. In the absence of energy, lipolysis can hydrolyse these fatty acids into free unsaturated fats, which enter the circulatory system and reach various parts of the body where they oxidise to generate energy [109,110]. In obese people, due to an overabundance of lipids, white adipocytes increase in size and number compared to the normal levels.

## 6. Effect of Mushroom Consumption on Gut Microbiota

The beneficial effects of edible mushrooms and their polysaccharides on the gut microbiota, which are closely linked with the body weight, are currently a major focus in the field. A study in mice reported that administering the concentrates of *G. lucidum* reduced the body weight by modifying the microbiota, suggesting that mushrooms might be used as a potential probiotic for weight reduction [40]. The effect of HFD on gut microflora is more pronounced than the effect on energy balance. HFD-induced changes in the gut microbiota have been shown to reduce Firmicutes to Bacteroides ratio, which is related to high energy accumulation, fat storage, and intestinal homeostasis over time. Through the provocative rundown and platelet markers, obesity negatively affects the immunity. Several studies have examined the anti-obesity effects of polysaccharides from various mushrooms in vitro and in vivo [87,103,111]. Polysaccharides from *Coriolus versicolor* initiated an immunomodulatory effect in mice splenocytes through the MAPK-NF-B pathway [112]. A polysaccharide from *Tremella fuciformis* hindered the differentiation of 3T3-L1 adipocytes by reducing the mRNA expression, suggesting that this polysaccharide could be a potential prebiotic for obesity [74]. Cure of adipocytes with *G. lucidum* diminished adipogenic record factor articulation, which increases glucose and lipid transport and activates AMPK pathway, suggesting its potential as an anti-obesity drug [113].

Being overweight could cause several other illnesses and result in a reduced lifespan. A recent study suggests that changes in the gut microbiota are associated with obesity and other related metabolic syndromes [114,115,116]. The gut microbiota comprises trillions of microorganisms that perform several functions, including nutrient metabolism, maintaining the gastrointestinal cells, modulating the immune system, protecting against the invasion of pathogens, and balancing the endotoxins. The gut microbiota generate energy from food and can cause overweight and T2DM. It has been observed that in overweight mice, the gut microbiota draws out more energy from food than lean mice [117]. In healthy people, vancomycin treatment for one week modifies the gut microbiota, which results in reduced insulin sensitivity [118]. Additionally, the transfer of gut microbiota of any lean person to an overweight person leads to the development of insulin sensitivity in the recipient. These results suggest that changes in gut microbiota could cause obesity and T2DM.

In HFD animals, the levels of proteins that play a role in maintaining tight junctions of the intestine are lower than those in chow-fed animals. Administration of *G. lucidum* extract could recover the levels of those proteins, which resulted in the maintenance of the integrity of the intestine and prevention of the translocation of pro-inflammatory endotoxins from gut bacteria to blood (for example, lipopolysaccharides) [40]. Using a mouse obese model, it has been observed that feeding of high-fat diet for eight weeks increased the body weight, liver weight, fat accumulation, and lipid deposition in hepatocytes and adipocytes compared to the control group that were fed with chow. Supplementation with the water extract of *G. lucidum* reduced the weight gain and accumulation of fats in HFD mice. *G. lucidum* also improved glucose tolerance and insulin sensitivity. Compounds in *G. lucidum* that reduce obesity are high molecular weight polysaccharides (greater than 300 kDa). Fungal polysaccharides cannot be digested in the stomach or small intestine. However, the large intestine can digest them and produces short-chain fatty acids, consequently secreting GLP-1. GLP-1 and short-chain fatty acids ultimately enter the blood and affect the brain, muscles, adipose tissues, and liver. Additionally, GLP-1 reduces gastric emptying and thereby, the appetite. It also reduces the deposition of fats, resistance to insulin, and inflammation. It also upregulates the proliferation and downregulates apoptosis in β-cells [40]. This suggests that *Escherichia coli* in the large intestine releases proteins that enhance or aid in the production of GLP-1 and peptide YY, which increases satiety [103]. These results indicate that the water extract of *G. lucidum* could be a potential prebiotic agent that can be used for the treatment of obesity and related complications [40]. Button mushrooms (*A. bisporus*) and *L. edodes* contain several polysaccharides, indicating their potential to stimulate the growth of beneficial bacteria in the gut.

*Hirsutella sinensis* is the asexual form of *Ophiocordyceps sinensis*. It modifies the composition of the gut microbiota and is beneficial in reducing obesity, inflammation, and diabetes in HFD mice. Table 2 presents the effects of various mushrooms on gut microbiota.

## 7. Mushrooms as Potential Anti-Obesity Agents

Numerous clinical studies have reported the anti-obesity effects of mushrooms. A clinical trial was conducted on 73 obese adults in which mushrooms were substituted for red meat as a part of the regular diet. At the end of one year, less energy intake, reduced body weight, low body mass index, low waist circumference, and low systolic and diastolic blood pressure was reported in the subjects on mushroom diet [136]. Few animal studies have also reported the anti-obesity effects of mushrooms. One study showed the anti-obesogenic effect of mushroom (*Grifola gargal*- 2%) after its administration in mice for 42 days. This study observed a reduction in blood glucose, triglyceride, and adipose tissue [105]. In another study, six weeks mice were divided into six groups: (1) low-fat diet control group, (2) low dose of mushroom (*G. lucidum*)- 100 mg/kg in the low-fat diet group, (3) high dose of mushroom (*G. lucidum*)- 300 mg/kg in the low-fat diet group, (4) high-fat diet control group, (5) low dose of mushroom (*G. lucidum*)- 100 mg/kg in the high-fat diet group, (6) high dose of mushroom (*G. lucidum*)- 300 mg/kg in the high-fat diet group. Mice in each group were divided into two cages, with three mice in each cage. The temperature was maintained at 25–28 °C. *G. lucidum* was administered once a day to each mouse for 12 weeks, and weight and food intake were monitored regularly. Weight was significantly reduced in the low-fat diet group [137].

## 8. Discussion

Epidemiological findings suggest that the intake of plant-based foods could have health benefits related to the incidence of T2DM, obesity, cardiovascular diseases, and some cancers. These effects have been attributed to the high content of fibres, phytonutrients, vitamins, and minerals found in these plant-based foods, in addition to their low content of saturated fat. Plant extracts or isolated phytochemicals and herbal concoctions are consumed as health supplements.

This review aims to provide an overview of the effects of edible mushrooms that exhibit anti-obesity effects. We emphasised the cellular and physiological mechanisms underlying the effects of mushrooms on obesity and highlight the effects related to the variation of hormones that regulate satiety, adipocyte function, and insulin sensitivity. The reports of previous studies discussed in this review suggest the potential impact of bioactive compounds in mushrooms in regulating the complications of obesity by the modulation of biochemical or cellular pathways. This review also focuses on the studies that reported the effectiveness of *P. ostreatus* intake in adults. The anti-obesity effects of all of the oyster mushrooms were investigated in a total of eight clinical trials.

Obesity is the most common global health challenge. It is a metabolic syndrome and its complications such as hypertension, atherosclerosis, T2DM, and dyslipidemia, are usually caused by an imbalance in energy expenditure, sedentary lifestyle, dietary habits, environmental factors, and behavioural factors such as tobacco.

Mushrooms are cholesterol-free and have a low-fat content. They contain selenium, ergothioneine, and other bioactive compounds such as terpenes, glycans, comatin, fibres, flavonoids, sterols, polyphenols, polysaccharides, alkaloids, and other highly beneficial nutrients including vitamins, minerals, and phytochemicals, which are similar to those present in vegetables. Mushrooms have been used for thousands of years as food and to treat several diseases.

Hunger and satiety are controlled by diverse neural and endocrine collaborations between the gut, brain, and adipose tissues. The hormone ghrelin, produced by the gastrointestinal tract when the stomach is empty, is believed to act on hypothalamic brain cells in the central nervous system. The presence of food in the gastrointestinal tract galvanises the vagus nerve of the afferent pathway prior to the inhibition of the hunger centre in the brain. Similarly, food intake induces the discharge of cholecystokinin by the epithelial cells of the small intestine, which alternatively inhibits the action of hunger-stimulating neuropeptide Y in the hypothalamus. Leptin is a satiety-inducing hormone released by adipocytes upon stimulation by insulin. Leptin hinders the action of neuropeptide Y and the hunger-stimulating fatty acid neurotransmitter anandamide and triggers the hunger-suppressing peptide α-melanocyte-stimulating hormone.

The anti-obesogenic medicine orlistat impedes the action of human pancreatic lipase by establishing a covalent bond with the enzyme at its catalytic site. Phytochemicals such as polyphenols and dietary fibres could reduce the cholesterol in bile acids formed by the liver. Bile is secreted into the small intestine to ease the digestion and absorption of dietary lipids. In enterohepatic circulation, bile acids are reabsorbed by enterocytes and transported back to the liver. When phytochemicals in the food mix with bile acids, they inhibit enterohepatic circulation and increase the excretion of bile acid through faeces. This could ultimately cause the reduction in blood cholesterol levels and have positive effect on the blood lipid profile.

Dietary fibres have a bulking effect upon ingestion that can influence satiety and interrupt gastric emptying, thereby reducing glycaemic index. Fibres generate less energy than carbohydrates. Some fibres are digested by fermentation in the large intestine and add to dietary energy intake. However, plant fibres can also obstruct the absorption of prescription drugs, although this can be prevented by following the prescription guidelines. We will now go through the fibres and phytochemical effects of mushrooms.

Mushrooms such as button mushrooms (*A. bisporus*) and shiitake (*L. edodes*) which have high polysaccharide content have been shown to promote the growth of beneficial gut bacteria. *H. sinensis* also alters the composition of the gut microbiota and has demonstrated anti-obesogenic, antidiabetic, and anti-inflammatory effects in HFD mice. Several substances, such as fibres and polysaccharides in mushrooms are beneficial to the human body via modulation of the gut microbiota without being directly absorbed by the body. These substances can act as prebiotics and are potential candidates for the development of antidiabetic and anti-obesogenic treatments.

Mushrooms significantly affect plasma blood sugar levels in the fasting state and at 2 h after breakfast. They could be included with vegetables in a hospital setup for patients with diabetes. One trial included men and women taking medication. Women were fed with cooked mushrooms in the place of vegetables along with the medication. However, men were provided with a powdered form. There was a significant decrease in fasting plasma glucose and Hba1 levels. These effects of mushrooms are due to the presence of a bioactive chemical, which is similar to that found in vegetables. This bioactive compound in the mushrooms protects B cells of the pancreas from dysfunction, which is caused by the pro-inflammatory cytokines. Mushrooms are rich in β-glycans which are responsible for the anti-inflammatory effect of mushrooms. They also increases the uptake of glucose by the peripheral tissue. In addition to the enhanced glucose absorption from the intestine, *P. ostreatus* consumption reduced glycogen synthase kinase levels and increased the secretion of insulin.

Diets that include mushrooms have a significant effect on lipid profiles. Low-density lipids and triglyceride levels were reduced in subjects with and without diabetes upon consumption of mushrooms. Similarly, blood pressure was also reduced upon mushrooms intake. Previous reports suggest that diastolic pressure increases upon consumption of vegetables. However, with mushrooms, it is reduced. Consumption of powdered form of *P. ostreatus* produces significant antioxidant activity of glutathione in red blood cells and glutathione peroxides in the plasma. Oxidised low-density lipid levels were reduced in test group patients with hyperlipidaemia upon the consumption of mushrooms. However, this change was not observed in the control group. In another study, it was reported that modification of gut microbiota was associated with the prevention of cardiovascular diseases. *G. lucidum* modifies the gut microbiota and the absorption of dietary polysaccharides. It stimulates the secretion of short-chain fatty acids when digested by bacteria in the large intestine. Short-chain fatty acids secrete GLP-1 from the enterocytes. They also enhance insulin sensitivity and intestinal integrity and reduce inflammation. Both GLP-1 and short-chain fatty acids enter the blood, modify the physiological mechanisms of different organs, and are associated with a decreased incidence of obesity. They also reduce lipid accumulation in the muscle and liver and reduce insulin resistance.

## 9. Recommendations and Implications for the Future

Different clinical trials have been conducted on mushrooms in various forms and their beneficial effects on health have been analysed. They include fresh, cooked, and powdered forms. This manuscript reviewed in vitro and in vivo studied on the anti-obesity effects of edible mushrooms by modulating gut microflora. The findings of the clinical trials suggest that edible mushrooms can be used as alternative to vegetables; they contain several bioactive compounds and could be used as nutraceuticals. They also contain essential nutrients such as vitamins and minerals and have low sodium and cholesterol contents. Therefore, it is an excellent alternative food source for patients with hypertension. They also contain trace elements such as selenium which aids in improving human health. Therefore, edible mushrooms are potential candidates for preventing obesity and several other chronic ailments.

## Figures and Tables

**Table 1:** Weight loss, anti-obesity, and hypolipidemic properties of mushrooms.

Name of Mushroom	Summary of Methods	Outcome of Study	References
*Tremella fuciformis*	Water-soluble fraction obtained by water extraction and polysaccharides from ethanol extraction	The differentiation of 3T3-L1 adipocytes was inhibited by mushroom	[74]
*Agaricus bisporus*	Equivalent amounts of mushroom fibre and sugar beet fibre-fed to rats for 4fourweeks; liver weight studied for both groups of rats	The cellulose powder group should higher HDL cholesterol concentration than the mushroom fibre group.	[75]
*Pleurotus geesteranus*	Exopolysaccharides were extracted from mushrooms and tested on diabetes-induced mice	The hypolipidemic impact of the polysaccharide explored in streptozotocin-prompted diabetic mice, diminished plasma glucose levels, all-out triacylglycerol and cholesterol focuses by 17.1%, 18.8%, and 12.0%	[76]
*Hericium erinaceus*	Mice were fed a high-fat diet along with extracts of Yamabushitake mushroom	A substantial diminution in increased body mass, fat weight, and triacyl-glycerol level in serum and hepatic were observed after 28 days of a high fat diet.	[77]
	Exobiopolymer extracted from mycelial culture of mushroom was studied on hyperlipidemic mice	A major reduction in the overall plasma cholesterol (32.9%), cholesterol (45.4%), Low-Density Lipoprotein (LDL) atherogenic index (58.7%), triglyceride (34.3%), phospholipid (18.9%), and hepatic HMG-CoA reductase activity (20.2%) was observed after administration of 200 mg/kg dose.	[78]
*Lentinula edodes*	The diet containing varying proportions of mushroom with a high-fat diet was fed to mice for 4 weeks against a normal diet and high-fat diet control.	The mRNA expression of cholesterol 7-α-hydroxylase 1 (CYP7A1) was reduced in hypercholesterolemic mice and amplified by eritadenine and *L. edodes* (5, 10, and 20%) supplementation. Treatments with eritadenine and L. edodes were shown to decrease lipid build-up in hepatic tissues.	[79]
	Hypercholesterolemia Albino rats were fed a diet containing fruiting bodies of mushrooms and checked for plasma and faeces biochemistry and liver histology.	A diet containing 5% *L. lepideus* fruiting bodies decreased total plasma cholesterol, triglyceride, LDL, total lipid, phospholipids, and LDL to HDL.	[80]
	Wister rats were fed a high-fat diet and mushroom extract for 30 days, and then biochemical parameters, including the stress markers, were determined.	*Lentinula edodes* decreased levels of glucose and urea. Lipid peroxidation was augmented in rats receiving the HFD, and *L. edodes* reduced malondialdehyde levels, thus preventing fatty acid oxidation.	[81]
*Lentinus lepideus*	Hypercholesterolemia Albino rats were fed a diet containing mushroom fruiting bodies and checked for plasma and faeces biochemistry and liver histology.	Total plasma cholesterol (TC), triglyceride (TG), LDL, total lipid, phospholipids, and the LDL/HDL ratio was decreased in hypercholesterolemic rats after a diet containing 5% *Lentinus edodes* fruiting bodies.	[59]
*Pholiota nameko SW-02*	The mice hyperlipidemic model was established to study the effects of mycelia zinc polysaccharide (containing zinc, glucose, mannose, galactose, and arabinose) on lipid profile and oxidative stress.	The supplementation of mycelia zinc polysaccharide might progress blood lipid levels (TC, TG, HDL-C, LDL-C, and VLDL-C), liver lipid levels (TC and TG), and antioxidant status.	[82]
*Pleurotus eryngii*	Hypercholesterolemia Albino rats were fed a diet containing fruiting bodies of mushrooms and checked for plasma and faeces biochemistry and liver histology.	Total plasma cholesterol (TC), triglyceride (TG), LDL, total lipid, phospholipids, and the LDL/HDL ratio was decreased in hypercholesterolemic rats after a diet containing 5% *Pleurotus eryngii* fruiting bodies	[83]
*Pleurotus ferulae*	Hypercholesterolemia Albino rats were fed a diet containing mushroom fruiting bodies and checked for plasma and faeces biochemistry and liver histology.	Supplementation with 5% *P. ferulae* fruiting bodies to hypercholesterolemic rat decreased low-density lipoprotein (LDL), total plasma cholesterol, triglyceride, total lipid, phospholipids, and LDL/high-density lipoprotein ratio by 71.15%, 30.02, 49.31, 30.23, 21.93, and 65.31%, correspondingly.	[84]
*Pleurotus ostreatus*	Hypercholesterolemia Albino rats were fed a diet containing fruiting bodies of mushrooms and checked for plasma and faeces biochemistry and liver histology.	Total plasma cholesterol, triglyceride, low-density lipoprotein (LDL), total lipid, phospholipids, And LDL/HDL ratio was reduced in hypercholesterolemic rats after 5% powder of *Pleurotus ostreatus* fruiting bodies.	[85]
*Pleurotus salmoneostramineus L. Vass*	Hyper and normo cholesterolemia rats were fed a diet containing fruiting bodies of mushrooms and checked for plasma and faeces biochemistry and liver histology.	*P. Salmoneostramineus* fruiting bodies (5% administration) in hypercholesterolemic rats reduced LDL/HDL ratio, total plasma cholesterol, triglyceride, LDL, total lipids, and phospholipids.	[86]
*Pleurotus tuber-regium*	Mushroom extracellular polysaccharides were orally administered to obese diabetes-induced mice for 8 weeks, and liver PPAR-α expression was studied.	Serum TG, LDL, and total cholesterol concentration were decreased, and HDL level was increased after *P. tuber-regium.*	[87]
*Pleurotus ostreatus*	Hypercholesterolemic Wistar rats were fed a 5% dried mushroom diet and studied for biochemical markers of cholesterol metabolism.	*Pleurotus ostreatus* administration reduced serum and liver cholesterol level, LDL production, cholesterol absorption, HMG-CoA activity in the liver, and redistribution of cholesterol in favor of HDL.	[88]
*Adiantum capillus-veneris L.*	Pharmacological modulation of pancreatic lipase and α-amylase/α-glucosidase studied using in-vitro and in vivo study on high cholesterol diet fed Wistar rats	*capillus-veneris* showed antiobesity and triacylglycerol-reducing effects compared to rats fed with a high cholesterol diet for 10 weeks.	[89]
*Aster spathulifolius Maxim*	Rats fed a diet with mushroom extract supplementation for 4.5 weeks were tested for hepatic and serum lipid levels.	*Aster spathulifolius* Maxim extract (ASE) treatment includes fat intake and lipogenesis-related genes. It also increases the level of phosphorylated AMPKα in obese rats.	[90]
*Kluyveromyces marxianus*	Hyperlipedimic rats were fed a diet supplemented with three different dosages of mushroom extract and measured for serum and hepatocyte lipid concentrations.	*K. Marxianus* administration significantly reduced serum and liver total cholesterol, triglyceride, LDL cholesterol, and atherogenic index in rats while HDL cholesterol level and the anti-atherogenic index were increased.	[91]
*Auricularia auricula-judae*	Rats were fed with high-fat diet along with mushroom extract. The impacts on preventing hepatic steatosis were studied. In vitro study was carried out for the mechanistic study of mice adipocytes	Plasma lipid and liver enzymes were reduced after supplementation of *Auricularia auricula-judae.*	[92]
*Collybia confluens*	The effects of three weeks of mycelial powder administration on plasma glucose and biochemistry were studied on diabetic mice.	TG and TC level in the liver was decreased by *Collybia confluens.* AST and ALT activity was also reduced.	[93]
*Cordyceps militaris SU-12*	The structure of residue polysaccharides of mushrooms was studied using gas chromatography. Rat study was carried out to see its impact on plasma lipid profile and anti-oxidant potential.	Residue polysaccharide reduced blood and liver lipid levels, improving glutamate pyruvate transaminase and antioxidant activity.	[94]
*Flammulina velutipes*	The effect of active components in the mushroom extract was studied through administration for eight weeks into diets of hamsters. The outcomes investigated included serum and liver lipid profiling.	*Flammulina velutipes* (3%) powder and extract reduced the concentration of TC, TG, LDL, and HDL in the serum and liver.	[95]
*Grifola frondosa*	The cholesterol-lowering effects of mushroom fibre were investigated after feeding the cholesterol-free supplemented diet for four weeks. Serum cholesterol concentration and LDL receptor mRNA were determined.	*Grifola frondosa* fiber depressed the serum total cholesterol level by augmentation of faecal cholesterol excretion.	[96]
*Auricularia polytricha*	anti-hypercholesterolemic effects of the mushroom extract on hypercholesterolemic mice models were studied.	The total cholesterol in the Soluble Polysaccharide *Auricularia polytricha* ingestion groups considerably reduced 34.6 ± 7.6% and 33.3 ± 7.9% with doses of 4.5 and 9.0 mg/kg BW on the 29th day.	[97]
*Ganoderma lucidum*	Invitro analysis of mushroom extracts was carried out to determine free radical scavenging potential. In vivo antioxidant potential was determined through blood levels of stress markers in mice fed with the supplemented diet. Cardiovascular risk factors were determined through serum lipid profiling of mice	Hot water extract at 200 mg/kg b.w. lowered plasma levels of total cholesterol, triacylglycerol, and LDL cholesterol and increased HDL cholesterol.	[98]
	Ergosterol peroxide potential to inhibit triglyceride synthesis was determined at protein and mRNA levels and through differentiation of 3T3-L1 adipocytes	The mitotic clonal expansion (MCE) stage blocked the phosphorylation of mitogen-activated protein kinases (MAPKs), which play a part in cell production and the stimulation of early differentiation transcription factors. Ergosterol peroxide also significantly reduced triglyceride production and differentiation in 3T3-L1 cells.	[38]
*Pleurotus eryngii*	Invitro analysis was performed on DPPH and hydroxyl radical scavenging potential. Three-week administration of supplemented diet on hyperlipidemic mice model was carried out to investigate the antiatherogenic potential (through lipid profiling and inflammatory enzyme markers)	Hepatic lipid accumulation was significantly reduced by *Pleurotus eryngii* administration.	[99]
*Echigoshirayukidake*	Feeding supplemented diet to rat models for 15 weeks on obesity (weight gain), and insulin resistance was investigated.	Supplementation to the eating routine altogether (*p* < 0.01) smothered the body weight gain and furthermore instinctive fat aggregation throughout the taking care of period contrasted with the control diet	[100]
*Ganoderma applanatum*	The effect of feeding diet supplemented with mushroom polysaccharides for two months on serum, and tissue lipid profile and weight gain were determined	Organization of *Ganoderma applanatum* remove at various portion levels essentially diminished the all-out cholesterol, TG, LDL, cholesterol levels, and the atherogenic file from 50 to 150 mg/kg body weight.	[101]
*Sparassis latifolia*	Six weeks trial through feeding the diet supplemented with the fruiting body of mushroom was carried out. Outcome measures were weight gain, food efficiency ratio and serum lipid profile.	Significantly suppressed the occurrence of non-alcoholic fat deposits in the liver	[102]
*Dictyophora indusiata*	The modulatory impact of mushroom polysaccharide on obese mice model fed a high-fat diet were determined through studying the lipid profile and inflammatory markers.	Bodyweight, adipocyte size, fat accumulation, adipogenic and liver-associated markers, glucose levels, endotoxin (Lipopolysaccharide, LPS) levels, and inflammatory cytokines were diminished significantly. Furthermore, the study exposed that *Dictyophora indusiata* polysaccharide treatment inverted the dynamic variations of the gut microbiome community by causing a decrease in the Firmicutes to Bacteroidetes ratio	[103]
*Flammulina velutipes*	Mushroom chitosan fed for five weeks to rats was tested for its effects on serum lipid profile, liver function enzyme markers, and weight gain.	Mushroom chitosan complex acted to stifle amplification of the liver from fat affidavit coming about due to a high-fat eating routine and re-establish hepatic capacity. The lipid content of dung indicated a stamped increment corresponded with the mushroom chitosan portion.	[104]
*Grifola gargal*	A human clinical trial was performed to study the effect of four weeks of feeding the mushroom extract on Triglyceride levels. The mice model was also used to study blood glucose, triglyceride, and adipose tissues.	Decreased blood glucose and fatty oil levels, and fat tissue. *Grifola gargal* (2.0 mg/mL) essentially stifled the expression of the cytokine interleukin-6 in 3T3-L1 cells contrasted and control cells.	[105]
*F. velutipes, H. marmoreus, L. edodes, G. frondosa and P. eryngii*	Lipid metabolism was investigated in mice fed with Japanese mushrooms.	Utilization advanced the corruption of lipids in instinctive fat and restricted the ingestion of food lipids. Also, the high-fat eating routine that took care of gathering exhibited higher convergences of phospholipids; some of them had odd-chain unsaturated fats.	
*Pleurotus eryngii*	Effect of feeding mushroom supplemented diet to mice models was investigated on obesity (adipose tissues and blood parameters) and gut microbiota (gene sequencing)	Serum all out cholesterol and LDL cholesterol levels diminished, and lipid and complete bile acids in dung expanded	[106]
*Ganoderma resinaceum*	The antiobesity effect of the biologically active component was determined using extensive spectroscopic analysis. In vitro analysis was also performed on brown adipocytes.	Resinacein S reduced lipid drops size by overseeing lipid absorption anyway didn’t impact the detachment of C3H10T1/2 cells. Resinacein S extended the assertion of brown and beige adipocytes markers and updated the activity of brown and beige adipocytes in isolated C3H10T1/2 cells.	[107]

**Table 2:** Effect of various mushrooms on gut microbiota.

Name of Mushroom	Effect on Gut Microbiota	References
*Pleurotus eryngii*	*P. eryngii* polysaccharides altered the abundance of SCFA producing gut bacteria	[106]
*Pleurotus sajor-caju*	Growth of SCFA producing bacteria was reduced, and *E.Shigella* was decreased by *Pleurotus sajor-caju*.	[113]
*Flammulina velutipes*	increase in lactic acid-producing bacteria (*Lactobacillus, Lactococcus, and Streptococcus*) and SCFA-producing bacteria (*Allobaculum, Bifidobacterium, and Ruminococcus*)	[119]
*Hypsizygus marmoreus*		
*Lentinusedodes*		
*Grifola frondosa*		
*Pleurotus eryngii*		
*Ganoderma lucidum*	*G. lucidum* enhanced SCFAs producing bacteria and abridged sulfate-reducing bacteria in a time-dependent manner	[120]
*Lentinula edodes*	LESDF-3 was found to stimulate the synthesisof Bacteroides	[121]
*Bulgaria inquinans*	increase of *Faecalibaculum* and Parabacteroides abundance and the decrease of *Allobaculum*, *Candidatus_Saccharimonas*, and *Rikenella* abundance at the genus level	[122]
*Ganoderma lucidum*	There was an increase in *Bacteroides/Firmicutes ratio, Clostridium clusters IV, XVIII, XIVa* (*Roseburia* spp.), *Eubacterium* spp.) SCFAs production bacteria, reduction in *Oscillibacter* spp. and *E. fergusonii*.	[40]
	Increase in *Alloprevotella, Barnesiella, Parabacteroides, Bacteroides, Bacteroidales S24-7 and Alistipe.* Decrease in *Blautia, Roseburia, and Enterorhabdus*.	[123]
	Increase in *Blautia, Bacteroides Dehalobacterium*, and *Parabacteroides,* *Decrease in Proteus, Aerococcus, Ruminococcus, and Corynebactrium*.	[124]
	Increase in *Alloprevotella, Prevotella, Ruminococcus and, Alistipes, Peptococcaceae, Alloprevotella, and Defluviitalea,;* Decrease in *Turicibacter, Clostridium XVIII and Phascolarctobacterium*.	[125]
*Grifola frondosa*	Increase in *Akkermansia muciniphila, Bacteroidetes/Firmicutes, Porphyromonas gingivalis, Lactobacillus acidophilus, Roseburia intestinalis, Tannerella forsythia, and Bacteroides acidifaciens*.	[124]
	*Increase in Barnesiella Helicobater, Intestinimonas, Defluvitalea, Flavonifractor and Paraprevotella and Ruminococcus.* *Decrease in Butyricicoccus, Clostridium-XVI, and Turicibacter.*	[126]
	*Increase in Alistipes.* *Decrease in Streptococcus, Enterococcus, Staphlococcus, and Aerococcus.*	[127]
	*An increase in Bacteroidetes/Firmicutes ratio increased the abundance of Oscillibacter, Defluvitalea, and Barnesiella.*	[128]
	*Increase in Intestinimonas and Butyricimonas.* *Decrease in Turicibacter and Clostridium XVIII.*	[129]
*Phellinus linteus*	Increase in *Lachnospiraceae-NK4A136, Roseburia, Prevotella Lachnospiraceae-UCG-006, Anaerotruncus, Blautia, Eubacterium_xylanophilum, Ruminiclostridium-9, and Oscillibacter*.	[130]
*Coriolus versicolor*	Increase in *Akkermansia muciniphila*	[131]
*Hericium erinaceus*	Increase *in Bifidobacterium, Coprococcus, Desulfovibrio, Lactobacillus, Parabacteroides, Prevotella; Decrease in Corynebacterium, Dorea, Roseburia, Ruminococcus, Staphylococcus, Sutterella*	[132]
*Ganoderma lucidum*	Increase in Firmicutes, Proteobacteria (Helicobacter), Rikenella; Decrease in Acinetobacter, Actinobacteria (*Arthrobacter, Corynebacterium*), Bacteroidetes (*Bacteroides, Parabacteroides, Prevotella*), *Blautia, Brevundimonas, Clostridium, Coprobacillus, Cyanobacteria, Facklamia, Jeotgalicoccus, Sporosarcina, Staphylococcus, Streptococcus*	[133]
*Boletus edulis*, *Boletus pinophilus*, *Boletus aureus* (Porcini), *Armillaria mellea(Honey fungus), Lactarius piperatus (blancaccio), Pleurotus eryngii (King oyster)*	Increase in *Bifidobacterium* and *Lactobacillus* genera	[134]
Cyclocybe cylindracea (poplar mushroom), *Hericium erinaceus*, *Pleurotus eryngii*, *Pleurotus ostreatus* (Oyster mushroom)	Increase in *Bifidobacterium* spp. *Faecalibacterium prausnitzii (Ruminococcaceae), Eubacterium rectale/Roseburia* spp.	[135]
*Flammulina velutipes* (Enoki), *Hypsizygus marmoreus*, (White beech mushroom), *Lentinula edodes* (Shiitake), *Grifola frondosa*, (Maitake) *Pleurotus eryngii*	Increase in *Allobaculum, Bifidobacterium, Ruminococcus, Lactobacillus, Lactococcus, Streptococcus*	[119]

## Data Availability

Not applicable.
